# Controversy: Is Benzalkonium Chloride Necessary in Antiglaucoma Drops?

**DOI:** 10.5005/jp-journals-10008-1115

**Published:** 2012-10-16

**Authors:** Y Louati, T Shaarawy

**Affiliations:** Clinical Fellow, University of Geneva Hospitals, Glaucoma Sector, Geneva Switzerland; Assistant Physician and Head, Department of Ophthalmology, University Hospitals of Geneva Glaucoma Sector, Geneva, Switzerland

**Keywords:** BAK, Ocular surface disease and glaucoma, Bioavailability, Preservatives in glaucoma medication, Preservative free glaucoma medication.

## Abstract

Medical therapy is the first-line option in glaucoma management, with benzalkonium chloride (BAC) being the most frequently used preservative in antiglaucoma medications. Its use is however, known to be associated with deleterious effects on the ocular surface. This review is an attempt to critically evaluate whether BAC really is indispensable for better bioavailability of antiglaucoma drugs and consequently, better IOP control.

**How to cite this article:** Louati Y, Shaarawy T. Controversy: Is Benzalkonium Chloride Necessary in Antiglaucoma Drops? J Current Glau Prac 2012;6(3):104-107.

## INTRODUCTION

Medical therapy using ocular drops is in most cases the first-line option in glaucoma management, with benzalkonium chloride (BAC) being the most frequently used preservative in glaucoma preparations. But increasing evidence of BAC toxicity on ocular surface has progressively led to the emergence of a whole new generation of BAC-free antiglaucoma medication whose efficacy is still questioned by many physicians, a majority of glaucoma patients throughout the world continuing to be prescribed BAC-preserved eye drops, with subsequent fierce debate on BAC necessity in ophthalmic drops.

The aim of the present review is to try to determine whether BAC really is useful/indispensable for better penetrance and better intraocular pressure (IOP) control based on updated literature and evidence.

### What is BAC?

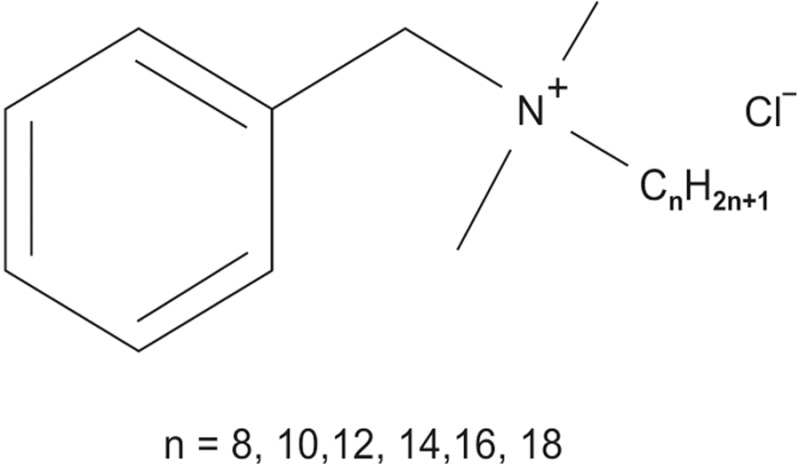



BAC is a nitrogenous cationic surface-acting agent belonging to the quaternary ammonium group that has an extremely wide range of applications with three major categories of use: As a biocide, as a cationic surfactant, as a phase transfer agent. BAC is used in skin antiseptics, hand sanitizers, high-level surgical instrument sterilizing and disinfection solutions, air and surface sprayable disinfectants, over-the-counter herpes cold sore and fever blister single-application treatments and eye and nasal drops as a preservative.

**Table 1:** **Table 1:** BAC concentration in antiglaucoma drops (%)

*Generic (Trade name)*		*Manufacturer*		*Preservative*	
Apraclonidine (Iopidine)		Alcon		0.01% BAC	
Betaxolol (Betoptic S suspension)		Alcon		0.01% BAC	
Bimatoprost (Lumigan 0.03%)		Allergan		0.005% BAC	
Bimatoprost (Lumigan 0.01%)		Allergan		0.02% BAC	
Bimatoprost/Timolol (Ganfort)		Allergan		0.005% BAC	
Brimonidine (Alphagan)		Allergan		0.005% BAC	
Brimonidine/Timolol (Combigan)		Allergan		0.005% BAC	
Brinzolamide (Azopt suspension)		Alcon		0.01% BAC	
Brinzolamide/Timolol (Azarga suspension)		Alcon		0.01% BAC	
Carteolol (Arteoptic)		Bausch & Lomb		0.005% BAC	
Dorzolamide (Trusopt)		MSD		0.0075% BAC	
Dorzolamide/Timolol (Cosopt)		MSD		0.0075% BAC	
Latanoprost (Xalatan)		Pfizer		0.02% BAC	
Latanoprost/Timolol (Xalacom)		Pfizer		0.02% BAC	
Levobunolol (Vistagan)		Allergan		0.005% BAC	
Timolol (Timoptic)		MSD		0.01% BAC	
Travoprost (Travatan)		Alcon		0.015% BAC	
Unoprostone (Rescula)		Novartis		0.015% BAC	

### BAC use in Ophthalmology

Bottled antiglaucoma topical medications contain numerous ingredients including the drug itself, its vehicle, a preservative, as well as other chemicals preventing the drug from binding to the inner surface of the plastic container. The need for sterility in multidose eye drops bottles requires the inclusion of an antimicrobial preservative. BAC is a highly effective preservative and the most commonly used in antiglaucoma medications with concentrations ranging from 0.004 to 0.025% (Table 1).

### Biological Activity

BAC biocidal activity is thought to be due to disruption of intermolecular interaction and dissociation of cellular membrane lipid bilayers which compromise cellular permeability control inducing leakage of cellular contents. BAC antimicrobial action is by means of dissolution of bacteria walls and membrane of their cellular contents that is unfortunately nonselective, exerting a toxic effect on human cells as well, even at low concentration (0.01%).^1,2^

### Safety Questioned

A proper ophthalmic preparation must ensure drug penetration into the globe, adequate efficacy, acceptable side effects and measures to prevent microbial conta-mination.^3^

Preservatives acting as detergents, such as BAC, not only help maintaining ophthalmic bottles sterility. They also disrupt the superficial lipid layer of the precorneal tear film allowing for subsequent evaporation of the aqueous layer that shortens break up time. They reduce the number of goblets cells resulting in failure of corneal epithelial wetting and thus, precorneal tear film thinning and malfunction, superficial punctate epithelial erosions and even ulcers, especially in patients with preexisting dry eye syndrome (DES). Swan^4^ for example found that repeated use of BAC at concentrations of 1:5,000 (0.02%) or stronger can denature corneal protein and cause irreversible damage to the eye (Table 2).

Even though DES is an age-associated condition like glaucoma itself with a reported prevalence of DES varying from 5.5. Up to 33.7%,^5-11^ symptoms of dry eye are reported in more than 60% of patients suffering from open-angle glaucoma suggesting higher incidence within this population.^30,32^ Described symptoms consist of foreign body sensation (31% in preserved group *vs* 14% in preserved-free), dry eyes (23% *vs* 14%), tearing (21% *vs* 14%), itchy eyelids (18% *vs* 10%), all of which are overall more frequent in patients taking preserved medications, observations that have been confirmed by very large-scale studies.^12,33^

Toxic and allergic conjunctivitis have also been reported with BAC use: Toxic effect by loss of contact between adjacent epithelial cells and cell death resulting from BAC insertion in cell membrane that reduces ionic resistance and increases water and ions influx leading to edema and cell damage, hence cell desquamation and ulcer.^3^ BAC also generates superoxide anions formation and immune inflammatory process involving Langerhaans cells that leads to reversible conjunctiva fibrosis^13^ that is associated with failure of glaucoma filtration surgery,^14-17^ because of excessive fibrotic postoperative wound healing induced by BAC.

Furthermore, studies have demonstrated that BAC use was associated with direct trabecular meshwork toxicity with significant cell death within 10 minutes of exposure to as little as 0.0001% BAC (1/100th of BAC concentration used in ophthalmic)^18^ leading to reduction of trabecular function and potentially worsening of the condition. These findings are of particular concern since we now know that trabecular meshwork cells within the meshwork were found to be statically lower in patients with primary open-angle glaucoma.^19^

BAC has also been incriminated in the development of cataract with higher incidence in the eyes exposed to preserved topical glaucoma therapy as compared to preservative free in a large prospective randomized study as well as to postoperative cystoid macular edema after cataract surgery.^20,21,34,35^

**Table 2:** **Table 2:** Frequency of symptoms reported by patients with preserved and preservative-free eyedrops at first visit^33^

		*Preserved eyedrops* *(n = 3469)*		*Preservative-free eyedrops* *(n = 552)*	
Discomfort upon instillation		43%		17%*	
Foreign body sensation		31%		14%	
Stinging or burning sensation		40%		22%*	
Dry eye sensation		23%		14%	
Tearing		21%		14%	
Eyelid itching		18%		10%	
Presence of symptoms of irritation between instillations		61%		36%	

*Preservative-free *vs* preserved comparison: p < 0.001 (χ^2^-test)

### Necessity Questioned

It has been suggested that through its detergent activity BAC facilitates drug penetration into the eye and thus enhances its efficacy. But many studies have now shown equal efficacy between same class preserved and preservative-free antiglaucoma medication and equal or improved tolerance^22-26^ making this past belief close to obsolete.

Besides industries have already developed alternatives to BAC, ranging from apparently less toxic preservatives (Polyquad, Purite, Sofzia…) to subtle mechanisms that already guaranty multi dose bottles sterility in antiallergic and lubricant preparations (ABAK^®^ COMOD^®^ antibacterial film, AADSTM^®^ silver coil combined with airless pump, VISMED^®^ system), through a whole new generation of preservative free single-dose units of antiglaucoma preparations.

**Table 3:** **Table 3:** Currently available preservative-free and BAC-free antiglaucoma preparations

Beta blockers: Timolol, carteolol, betaxolol, levobunolol		Miotic: Pilocarpine	
Carbonic anhydrase inhibitors: Dorzolamide		Alpha-agonists: Apraclonidine, brimonidine	
Combination: Cosopt (dorzolamide + timolol)		Prostaglandins: Tafluprost, travoprost	

### Alternatives to BAC

Clinical studies have now demonstrated that preservative-free formulations of antiglaucoma medications have the same efficacy as preserved formulations, achieving equivalent reductions of intraocular pressure (Table 3).^23-26^

Besides, Jong et al reported that switching patients with glaucoma from preserved to preservative-free medication reduced the permeability of the corneal epithelium, suggesting improvement in epithelial function.

Ammar et al^20^ demonstrated that substitution of BAC from topical ophthalmic drugs results in greater viability of cultured trabecular meshwork cells, suggesting better trabeculum meshwork function in patients in whom aqueous outflow is already compromised.

A recent large European Study assessed ocular symptoms in a total of 9,658 patients before and after switching from preserved to preservative-free eyedrops and demonstrated that stinging or burning sensation occurred in 48% of patients receiving preserved eyedrops compared with only 20% of those who received preservative-free eyedrops, whereas dry eye sensation was reported in 35 and 16% of the 2 groups, respectively. Similar reductions in the incidence of reported symptoms occurred in patients who reduced their exposure to benzalkonium.^12^

### Perspective

Studies have already shown solid evidence that commercially available preservative-free antiglaucoma formulations do offer clinical benefits to patients in term of safety and efficacy. Care should therefore be taken from now on to avoid long-term use of preservatives when possible, single dose units manufacturing and packaging still make them expensive and more difficult to use as compared to multiple dose bottles especially for older patients (hand arthritis for instance). Otherwise preparations with less toxic preservative should be developed especially for patients with the greatest exposure to high doses and/or prolonged treatments, for those suffering from preexisting ocular surface disease and those experiencing side-effects related to the ocular surface because of their current treatment.

Preservative-free drops emergence represents a real hope for global improvement in glaucoma patients care because of equally efficacious, less toxic and therefore more tolerated treatment possibilities, as compared to preserved drops, and thus increased likelihood of adherence to the treatment prescribed, all of which is responsible for better visual health, better quality of life and less use of health care resources.^3,12,14,26-31^
